# Integrated multi-omics analysis reveals variation in intramuscular fat among muscle locations of Qinchuan cattle

**DOI:** 10.1186/s12864-023-09452-9

**Published:** 2023-07-01

**Authors:** Hengwei Yu, Jianfang Wang, Ke Zhang, Gong Cheng, Chugang Mei, Linsen Zan

**Affiliations:** 1 College of Animal Science and Technology, Northwest A&F University, Yangling, 712100 Shaanxi China; 2 College of Grassland Agriculture, Northwest A&F University, Yangling, 712100 China; 3 National Beef Cattle Improvement Center, Yangling, 712100 China

**Keywords:** Cattle, Intramuscular fat, Transcriptomics, Metabolomics, Meat

## Abstract

**Background:**

Intramuscular fat (IMF) is closely related to the tenderness, marbling, juiciness, and flavor of meat. We used a combined transcriptome and metabolome analysis to investigate the molecular mechanisms underlying phenotypic variation among Qinchuan cattle.

**Results:**

The IMF content was relatively high in the meat of Qinchuan cattle bulls and differed among muscle locations, namely the high rib (15.86%), ribeye (14%), striploin (10.44%), and tenderloin (8.67%). *CCDC80* and the *HOX* gene cluster may regulate intramuscular adipose tissue deposition. Moreover, erucic acid (EA) was found to be the main metabolite in Qinchuan beef cattle, with a high concentration in IMF. The deposition of IMF could be regulated by the metabolic pathway for unsaturated fatty acids involving EA and the *ACOX3*, *HACD2,* and *SCD5* genes. In addition, differentially expressed genes and metabolites were enriched in three major KEGG pathways: purine metabolism, pyrimidine metabolism, and the metabolism of glycine, serine, and threonine.

**Conclusions:**

We identified a significant metabolite, EA, with variation in IMF. Its closely related genes, *ACOX3*, *HACD2*, and *SCD5,* co-regulate the metabolism of unsaturated fatty acids, ultimately affecting the accumulation of intramuscular adipose tissue in Qinchuan cattle. Consequently, Qinchuan cattle are an elite cultivar for high-quality beef production and have great potential for breeding.

**Supplementary Information:**

The online version contains supplementary material available at 10.1186/s12864-023-09452-9.

## Background

Cattle provide high-quality protein and therefore play a vital role in food and nutrition security. There is demand for high-quality beef, irrespective of price, and the intramuscular fat (IMF) content is one of the prominent indicators of meat quality [1]. Recent research has shown that the IMF content is strongly correlated with fatty acid types and contents [2]. IMF is a polygenic trait. Joint multi-omics analyses of the phenome, transcriptome, and metabolome have recently been utilized to explore the molecular mechanisms underlying complex traits [3]. Myristic acid, margaric acid, and trans-monounsaturated fatty acid, for example, are associated with IMF levels [4]. Hexanal and 1-octen-3-ol were identified as the major metabolites of volatile organic compounds in local Chinese chicken varieties [5]. A difference in odor between Hu sheep with low and high IMF contents may be related to the fatty acid profiles of triglycerides and diglycerides in the psoas major muscles [6]. Functional genes (*PNPLA3*, *PLIN1*, *PRKG1*, *TRIB3*, and *CREB5*) and differential metabolites (arachidonic acid and triglyceride) involved in lipid metabolism were associated with the IMF content in Enshi black pigs [3]. These omics analyses contribute to our understanding of the complex regulatory mechanism of muscle IMF.

The IMF content accounts for 15% of the variation in beef palatability [7]. Increasing the IMF content has been shown to improve the palatability of Hanwoo beef, thereby improving sensory sensitivity, flavor, and juiciness [8]. Lipid deposition in animals is primarily influenced by the breed and feed [9]. However, the location and function of muscle can also influence the IMF content [10]. In 18-month-old Alentejana and Barrosã cattle, the IMF content in semitendinosus muscle was lower than that in *longissimus* lumborum muscle [10]. At 24 months of age, the IMF content of *longissimus* muscle in Japanese Black steers (23.3%) was much higher than those of European bulls (Holstein–Friesian 4.7%, German Angus 4.4%, Belgian Blue 0.6%) [11]. At the age of 26 months, the IMF content of *longissimus* muscle in Japanese Black steers (34.3%) was significantly higher than that in Holstein steers (20.4%) [12].

Qinchuan cattle is a representative indigenous Chinese breed, characterized by delicious meat with a unique flavor. Under the influence of traditional Chinese food culture, there is a preference for local yellow beef for cooking, particularly for typical dishes, braised beef, and potatoes. Despite the large number of studies of IMF deposition, differences in IMF contents between muscle locations have not been determined. Therefore, in the present research, Qinchuan cattle were used as research subjects to evaluate variation in IMF contents and the underlying molecular mechanisms. In particular, four portions of meat were taken to determine the IMF content, namely tenderloin (psoas major muscle), striploin (*longissimus* lumborum muscle), high rib (chuck), and ribeye. mRNA and metabolite sequencing were used to identify differences among the four meat types with differential IMF contents. The aim of this study was to investigate biomarkers of IMF and the relationship between IMF and metabolite and mRNA levels in meat. Analyses of the molecular mechanism underlying IMF deposition in various meat types are of great value for the development and utilization of cattle resources.

The results of the present study were similar to those of these previous studies.

## Results

### Phenotypic variation in the IMF content among muscle locations

**Fig. 1:**
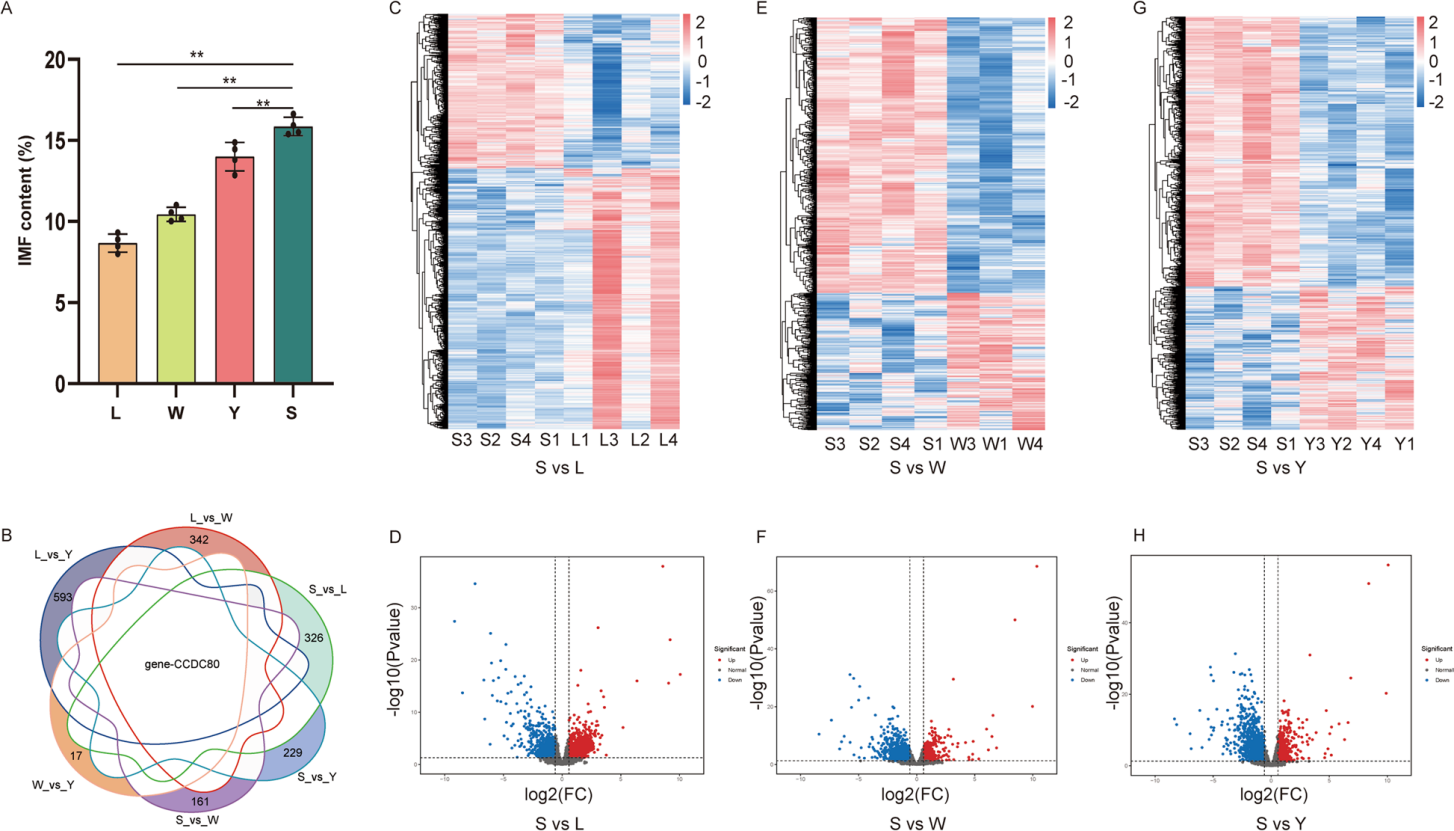
Basal analysis of transcriptome and phenotype profiles for IMF (*n* = 4). **A** Comparison of IMF contents in four groups, namely tenderloin (L group), striploin (W group), high rib (S group), and ribeye (Y group) (**P < 0.05). **B** Venn diagram showing DEGs in pairwise comparisons between the four groups. Heatmap of expression correlations between samples: S vs. L (**C**), S vs. W (**E**), and S vs. Y (**G**). Volcano plots of DEGs between samples: S vs. L (**D**), S vs. W (**F**), and S vs. Y (**H**)

The IMF content was evaluated in meat samples from four muscle locations of four 24-month-old bulls. There were four groups: tenderloin (L group), striploin (W group), high rib (S group), and ribeye (Y group). As shown in a histogram in Fig. 1A, there were statistically significant differences among groups (*P* < 0.01). The IMF contents from high to low were 15.86 ± 0.48% in the high rib, 14 ± 0.76% in the ribeye, 10.44 ± 0.38% in the striploin, and 8.67 ± 0.48% in the tenderloin. These results show that Qinchuan cattle is an informative resource for the production of meat with a high IMF content.

### DEGs and analysis of transcriptome data

The difference in IMF deposition between S and the other three muscle types was further explored at the molecular level. Transcriptome sequencing was used to filter DEGs related to the IMF content in various muscle locations of Qinchuan cattle. An RNA-seq analysis generated 98.58 Gb of clean data with Q30 greater than 90.75% for 15 samples (Table S1). The mapping ratios for clean reads were between 95.51% and 97.57% (Table S2). Changes in gene expression were evaluated based on FPKM values in various samples (Table S3). A principal components analysis (PCA) showed that the samples could be roughly divided into four groups, indicating that samples within each group had similar gene expression patterns (Fig. S1C). Pairwise comparisons of the four groups revealed only a single common DEG, *CCDC80* (Fig. 1B). There were 3031 DEGs in S vs. L, including 1901 up-regulated DEGs and 1130 down-regulated genes (Table S4, Fig. 1C and D), 1747 DEGs in S vs. W group, including 580 up-regulated genes and 1167 down-regulated genes (Table S5, Fig. 1E and F), and 739 up-regulated genes and 1393 down-regulated genes in S vs. Y (Table S6, Fig. 1G and H).

**Fig. 2:**
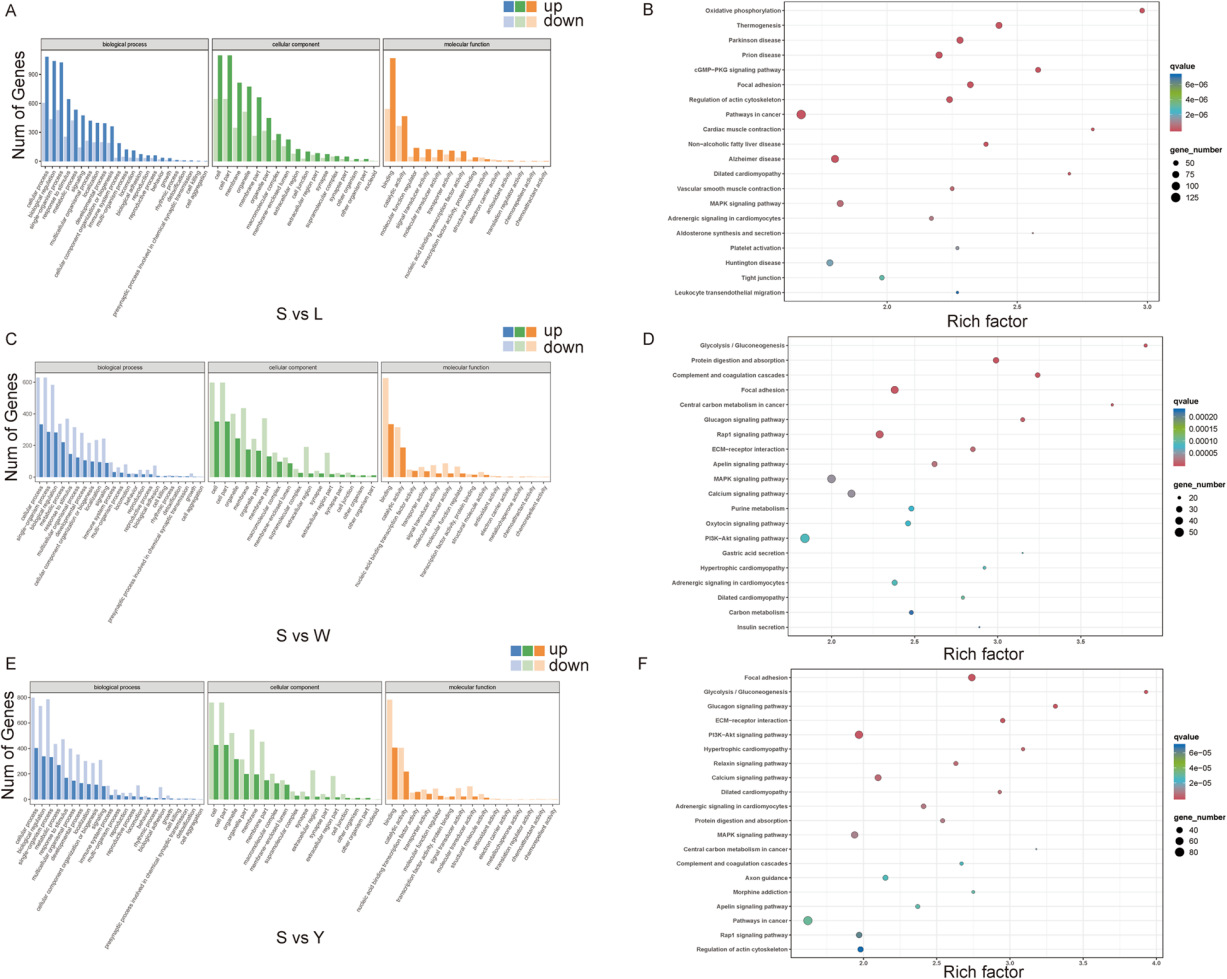
GO annotation analysis and KEGG pathway enrichment analysis of DEGs. Histogram of GO results for DEGs in S vs. L (**A**), S vs. W (**C**), and S vs. Y (**E**). Plot of the degree of KEGG pathway enrichment of DEGs for S vs. L (**B**), S vs. W (**D**), and S vs. Y (**F**). The vertical coordinate indicates the enriched pathway; the horizontal coordinate indicates the value of the enrichment factor (ratio of annotated DEGs to all genes in the enriched pathway)

In a GO analysis of DEGs in S vs. L, enrichment for 58 subcategories was detected, including 23 biological process (BP) terms, 17 cellular component (CC) terms, and 18 molecular function (MF) terms. In particular, 1689 genes were related to cellular processes in BP, 1744 genes were annotated to the cell and cell part process in CC, and 1611 genes were located within the binding portion in MF (Table S7, Fig. 2A). A KEGG enrichment analysis revealed that these DEGs were primarily involved in thermogenesis, cGMP − PKG, and MAPK signaling pathways (*P* < 0.05; Table S8 and Fig. 2B). In S vs. W, the DEGs in were also assigned to 58 subcategories, consisting of 23 BP terms, 17 CC terms, and 18 MF terms. There were 864 genes related to the biological regulation category in BP, 948 genes annotated to cell and cell parts in CC, and 959 genes associated with the binding portion in MF (Table S9, Fig. 2C). A KEGG enrichment analysis showed that the DEGs were primarily involved in the digestion and uptake of proteins, glycolysis/gluconeogenesis, MAPK, and PI3K − Akt signaling pathways (*P* < 0.05; Table S10 and Fig. 2D). In S vs. Y, the DEGs were also categorized into 58 subcategories, consisting of 23 BP terms, 17 CC terms, and 18 MF terms. Among them, 1199 genes were associated with cellular processes in BP, 1187 genes were associated with the cell and cell part process in CC, and 959 genes were located within the binding portion in MF (Table S11, Fig. 2E). A KEGG enrichment analysis showed that DEGs were primarily involved in glycolysis/gluconeogenesis, PI3K − Akt, MAPK, and the Apelin signaling pathway (*P* < 0.05; Table S12 and Fig. 2F). Based on these results, MAPK is a potential key signaling pathway in IMF deposition.

### DEMs and analysis of metabolome data

**Fig. 3:**
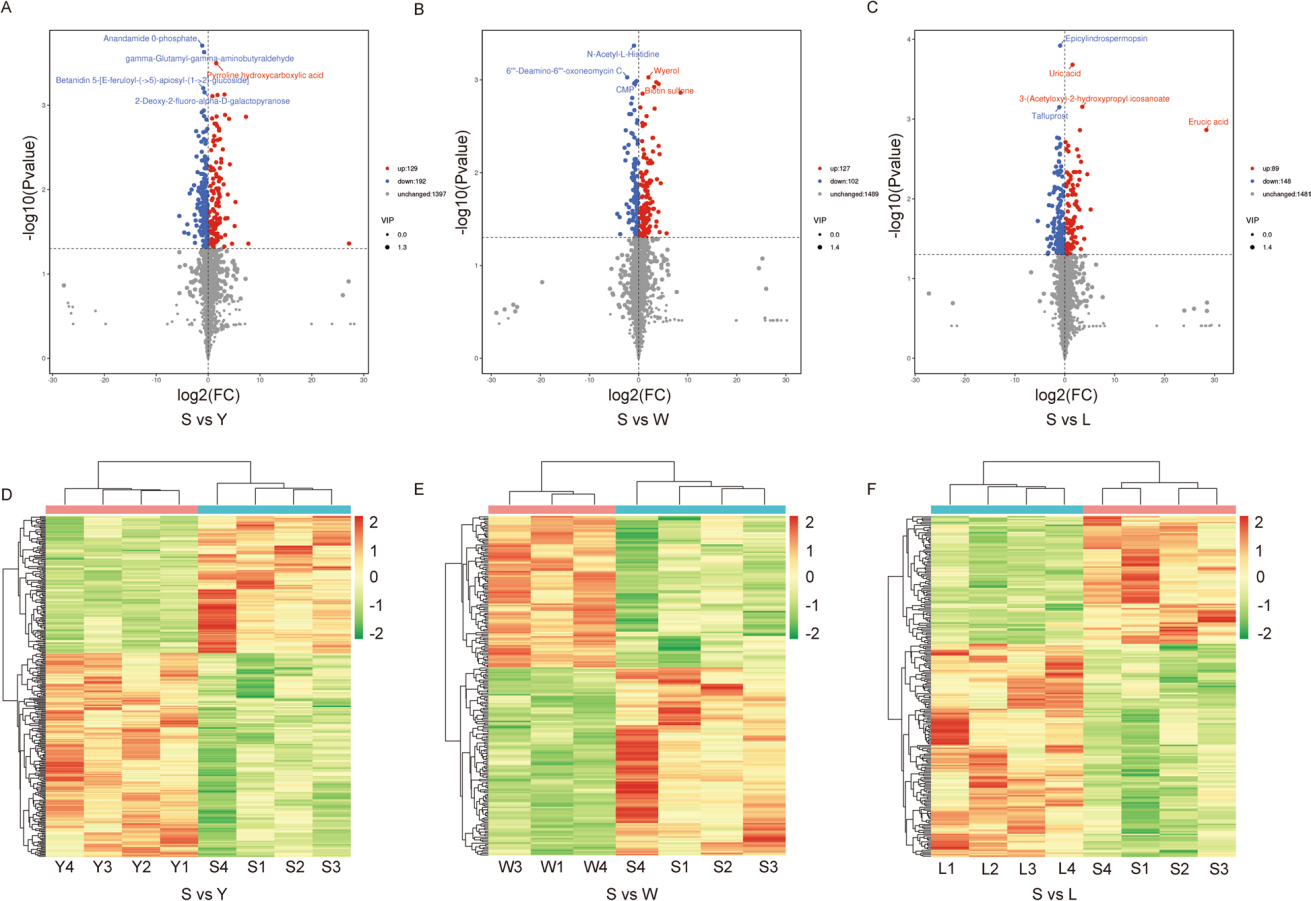
Heatmap and volcano plots of DEMs between samples. Heatmap of expression correlations between samples: S vs. Y (**A**), S vs. W (**B**), and S vs. L (**C**). Volcano plots of DEMs between samples: S vs. Y (**D**), S vs. W (**E**), and S vs. L (**F**)

**Fig. 4:**
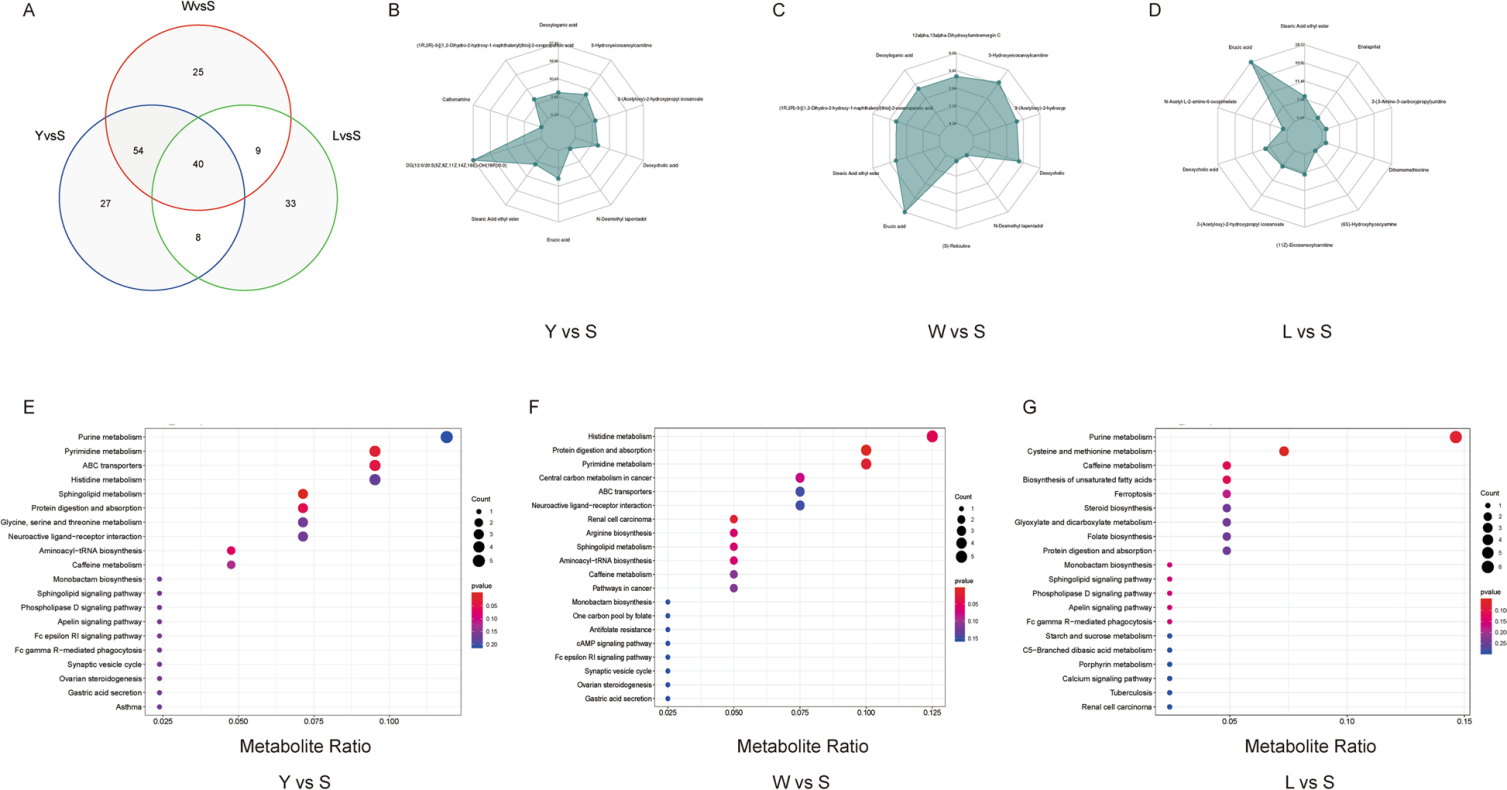
Venn diagram, radar analysis, and KEGG pathway enrichment analysis of DEMs. **A** Venn diagram of up-regulated DEMs for S vs. L, S vs. W, and S vs. Y. Radar diagram of DEMs between samples: Y vs. S (**B**), W vs. S (**C**), and L vs. S (**D**). The corresponding ratio was calculated for a quantitative analysis of DEMs, and the top 10 metabolites with the largest absolute value of log2FC were selected for visualization in the radar chart. The degree of KEGG pathway enrichment is plotted for DEMs for Y vs. S (**E**), W vs. S (**F**), and L vs. S (**G**)

A qualitative metabolome analysis of 15 samples was performed using the LC-QTOF platform. A total of 5,749 peaks were detected and 1,718 metabolites were annotated in positive and negative ion modes. In a PCA, PC1, PC2, and PC3 cumulatively explained 47.17% of the variance among groups (Fig. S1B). The within-group Spearman correlation coefficients were close to 1.0, suggesting that the DEM analysis method was reliable (Fig. S1C). In an orthogonal projections to latent structures-discriminant analysis (OPLS-DA) for the pairwise comparisons between S, W, L, and Y, Q^2^ values for all comparisons were greater than 0.75, indicating that the constructed model was appropriate (Fig. S1D–F). Metabolites were annotated against the KEGG database (Kyoto Encyclopedia of Genes and Genomes), HMDB (Human Metabolome Database), and LIPID MAPS (Lipid Metabolites and Pathways Strategy) (Fig. S2A–C). Then, DEMs were filtered based on the following criteria: |log2(fold change)|≥ 1 and VIP ≥ 1. In S vs. Y, 129 DEMs had a higher abundance in the S group than in the Y group, including DG (12:0/20:5(5Z,8Z,11Z,14Z,16E)-OH (18R)/0:0) and erucic acid (EA), whereas 192 DEMs were significantly less abundant in group S (Fig. 3 A and Table S13). In W vs. S, 127 DEMs were more abundant in the S group than in the W group, including EA, deoxycholic acid, and deoxyloganic acid, whereas 102 DEMs were much less abundant in group W (Fig. 3 B and Table S14). In L vs. S, 89 DEMs were more abundant in the S group than in the L group, while 148 DEMs were much less abundant in the L group (Fig. 3 C and Table S15). Similarly, the heatmap also depicted the same distribution of DEMs with groups (Fig. 3D–F). In comparison to the W, L, and Y groups, 40 common DEMs were upregulated in the S group (Fig. 4A and Table S16), and EA, carcinine, thiostatin, and glutaminylglutamic acid showed the greatest difference between the two groups (Fig. 4B–D). Then, a KEGG enrichment analysis was utilized to explore the biological mechanisms associated with phenotypic changes. In the Y vs. S comparison, the majority of DEMs were involved in 42 pathways related to purine metabolism, pyrimidine metabolism, histidine metabolism, sphingolipid metabolism, and glycine, serine, and threonine metabolism (Fig. 4E and Table S17). In W vs. S, the majority of DEMs were significantly involved in 40 pathways related to histidine metabolism, protein digestion and absorption, pyrimidine metabolism, and sphingolipid metabolism (Fig. 4F and Table S18). With respect to L vs. S, the majority of DEMs were involved in 41 pathways related to purine metabolism, cysteine and methionine metabolism, biosynthesis of unsaturated fatty acids, glyoxylate and dicarboxylate metabolism, and protein digestion and absorption (Fig. 4G and Table S19). In short, the unsaturated fatty acid metabolism pathway involving EA was extremely likely to be related to variation in IMF deposition.

### WGCNA of transcriptomics and metabolomics data

**Fig. 5:**
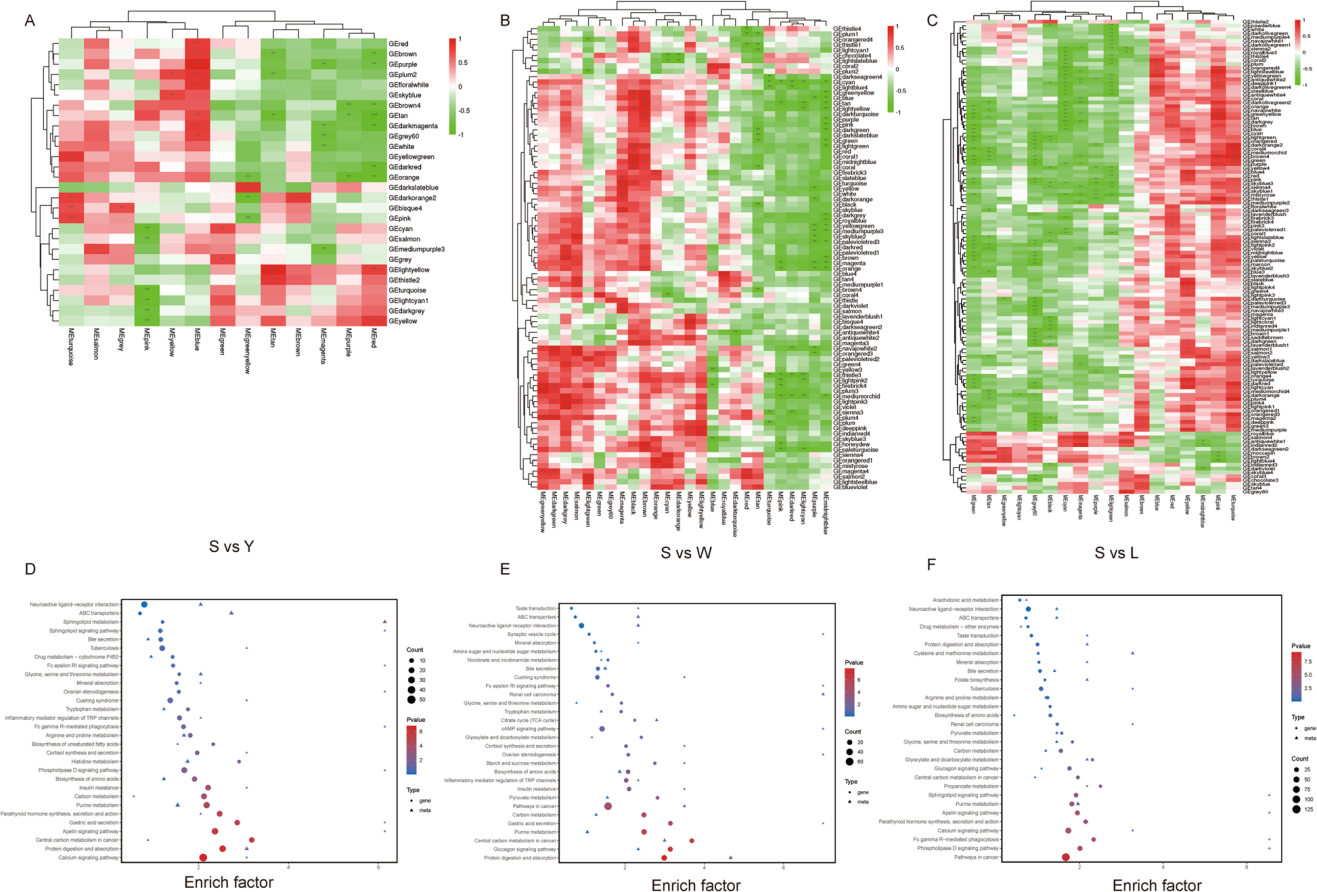
WGCNA and KEGG pathway enrichment analyses of transcriptome and metabolome data. WGCNA of transcriptome and metabolome between samples: Y vs. S (**A**), W vs. S (**B**), and L vs. S (**C**). The gene module is indicated on the right, the bottom shows the metabolite module, and the left and top show the clustering dendrograms of genes and metabolites. The closer the absolute value is to 1, the higher the correlation. Red indicates a positive correlation, while green indicates a negative correlation, with darker colors indicating stronger correlations. Asterisks indicate a significant correlation between metabolites and genes; *P < 0.05, **P < 0.01, and ***P < 0.001. KEGG pathway enrichment analysis: Y vs. S (**D**), W vs. S (**E**), and L vs. S (**F**)

Joint metabolome-transcriptome analyses can, to some extent, overcome the limitations of single omics research, providing more details regarding the transcriptional regulation of metabolic pathways. We performed a dimensionality reduction analysis, weighted correlation network analysis (WGCNA) [13] using transcriptome and metabolome data. Genes and metabolites were partitioned into different modules, and correlations between the modules were evaluated. In L vs. S, metabolites were clustered into 13 modules (ME) and genes into 28 modules (GE) (Fig. 5A). The correlation coefficient between the GEpurple module and the MEblue module was the highest (*r* = 0.95, *P* < 0.05). The MEblue module contained 183 metabolites, such as 3'-hydroxyropivacaine and l-malic acid. The GEpurple module contained 204 genes, such as *ATG4B*, *B3GALT6,* and *CACFD1* (Tables S20 and S21). In W vs. S, metabolites were clustered into 26 modules and genes into 87 modules (Fig. 5B). The correlation coefficient between the GEbisque4 module and the MEyellow module was the highest (*r* = 0.98, *P* < 0.05); the MEyellow module contained 91 metabolites, such as niridazole, 3-hydroxy-3-methylglutaric acid, and dihydroactinidiolide, and the GEbisque4 module contained 86 genes, such as *BRD2*, *FKBP5,* and *MECP2* (Tables S22 and S23). In L vs. S, metabolites were grouped into 18 modules and genes into 123 modules (Fig. 5C). The correlation coefficient between the GEplum4 module and the MEturquoise module was the highest (*r* = 0.93, *P* < 0.05); the MEturquoise module contained 265 metabolites, such as l-lysine, d-xylonate, and formyl phosphate, and the GEplum4 module contained 86 genes, such as *ACTR1B*, *FOXP1,* and *MAGOH* (Tables S24 and S25). Regarding the Y vs. S comparison, a KEGG analysis revealed enrichment for several important biological pathways, such as the sphingolipid signaling pathway, glycine, serine and threonine metabolism, phospholipase D signaling pathway, biosynthesis of amino acids, and protein digestion and absorption (Table S28 and Fig. 5D). In W vs. S, a KEGG analysis revealed enrichment for various biological pathways, such as amino sugar and nucleotide sugar metabolism, nicotinate and nicotinamide metabolism, glycine, serine and threonine metabolism, glyoxylate and dicarboxylate metabolism, and protein digestion and absorption (Table S29 and Fig. 5E). Regarding the L vs. S comparison, a KEGG analysis indicated enrichment for several essential biological pathways, such as arachidonic acid metabolism, protein digestion and absorption, folate biosynthesis, arginine and proline metabolism, and biosynthesis of amino acids (Table S30 and Fig. 5F). In conclusion, the enriched pathways associated with these genes and metabolites were related to unsaturated fatty acid metabolism and amino acid metabolism.

### Joint analysis of transcriptomics and metabolomics data

The top four DEMs were shared in three groups, including deoxyloganic acid, deoxycholic acid, ethyl ester of stearic acid, and erucic acid (Table S26). Compared with those in other groups, the abundance of erucic acid was highest among all differential metabolites in the S group and was related to the biosynthesis of unsaturated fatty acids (Fig. S2D). All gene-metabolite correlations were calculated based on the Pearson correlation method, and screening was performed according to the correlation coefficient (CC) and correlation coefficient *p*-value (CCP). Values of |CC|> 0.80 and CCP < 0.05 were selected as thresholds. The expression levels of the *HOXA* gene family (*HOXA5–7*, *HOXA9–10*), *HOXC* gene family (*HOXC6*, *HOXC8–10*), *HOXD* gene family (*HOXD1*, *HOXD4*, *HOXD8–9*), and *MYH1*, *SLC27A6,* and *CACNA2D*2 were higher in S than in other groups, suggesting that these genes have significant effects on the IMF phenotype with positive impacts (Fig. S2E). To gain insight into the relationship between genes and metabolites, a KEGG analysis of the co-enrichment of DEGs and DEMs revealed three critical pathways, purine metabolism (ko00230), pyrimidine metabolism (ko00240), and glycine, serine and threonine metabolism (ko00260) (Table S27).

**Fig. 6:**
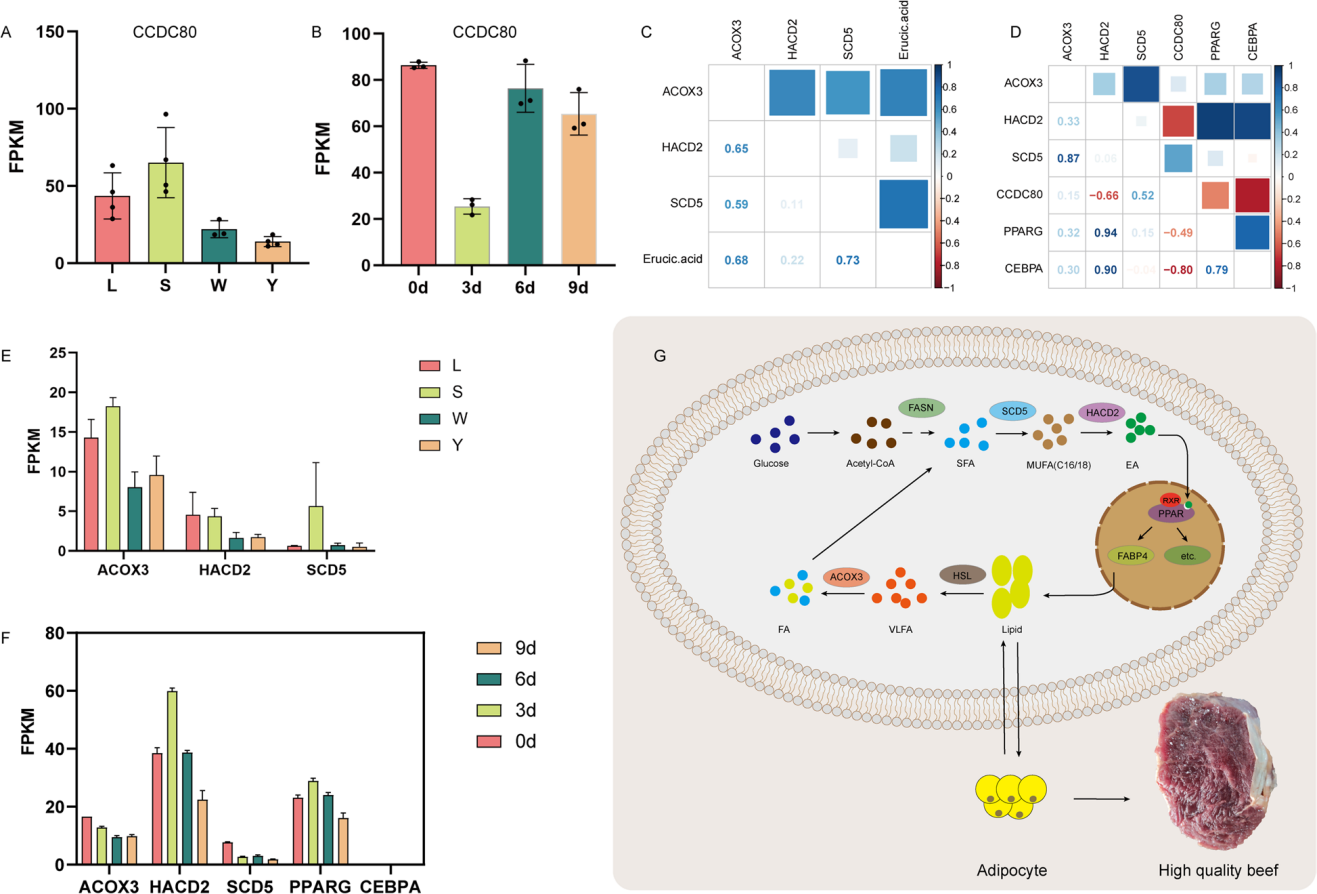
Potential molecular mechanism underlying intramuscular fat (IMF) regulation. **A** Levels of *CCDC80* expression in four muscle locations. **B** Levels of *CCDC80* expression on days 0, 3, 6, and 9 of intramuscular adipocyte differentiation. **C** Correlation between the EA content and *ACOX3*, *HACD2*, and *SCD5* expression levels in four muscle locations. **D** Correlations between the expression levels of *ACOX3*, *HACD2*, *SCD5*, *CCDC80*, *PPARG*, and *CEBPA* on days 0, 3, 6, and 9 of intramuscular adipocyte differentiation. **E** Expression levels of *ACOX3*, *HACD2*, and *SCD5* in four muscle locations. **F** Expression levels of *ACOX3*, *HACD2*, *SCD5*, *PPARG*, and *CEBPA* on days 0, 3, 6, and 9 of adipocyte intramuscular differentiation. **G** Potential regulatory mechanism by which the EA-involved unsaturated fatty acid synthesis pathway contributes to the formation of IMF

The bioinformatics analysis showed that EA levels are closely related to levels of *ACOX3*, *HACD2,* and *SCD5*. We further evaluated correlations between the EA content and levels of *ACOX3*, *HACD2,* and *SCD5* at four muscle locations. EA had the highest correlation with *SCD5* (*r* = 0.73), followed by *ACOX3* (*r* = 0.68) and *HACD2* (*r* = 0.22) (Fig. 6C and E). In an analysis of gene expression during intramuscular adipocyte differentiation, correlations were detected between *ACOX3* and *SCD5* (*r* = 0.87), *HACD2* and *PPARG* (*r* = 0.94), and *HACD2* and *CEBPA* (*r* = 0.9) (Fig. 6D and F). Based on the above findings, IMF deposition is associated with metabolic pathways for unsaturated fatty acids, including *ACOX3*, *HACD2,* and *SCD5*.

## Discussion

In general, the IMF content in the muscles of 24-month-old Qinchuan cattle bulls was relatively high, with differences among muscle locations as follows: high rib (15.86%), ribeye (14%), striploin (10.44%), and tenderloin (8.67%). Therefore, Qinchuan cattle can be used as a choice breed for the production of high-grade beef and has potential for improvement.

In this study, many DEGs related to variation in the IMF content have been reported previously, such as *PPARG*, *FAS*, *FABP3,* and *ELOVL5*. *PPARG* plays an essential role in adipose development and is an early factor involved in preadipocyte differentiation [14]. Single nucleotide polymorphisms (SNP) in *FAS* are associated with adipogenesis [15]. A close relationship has been detected between *FABP3* SNPs and the intramuscular fat content in Qinchuan cattle [16]. *ELOVL5* plays a vital role in the promotion of fatty acid synthesis [17, 18]. Of note, *CCDC80* (*DRO1*) was up-regulated in the high-IMF groups compared to the low-IMF group (Fig. 6A). Combined with mRNA sequencing data for intramuscular adipocytes on days 0, 3, 6, and 9 of differentiation in our laboratory [19], we found significant changes in the expression of *CCDC80* (Fig. 6B). The third day of adipocyte differentiation is a critical period, at which point the expression of this gene suddenly decreases, indicating that *CCDC80* may play an important regulatory role in fat deposition. *CCDC80* plays a key regulatory role in major physiological processes, such as weight control, energy metabolism, and apoptosis, and is closely linked to related diseases, such as obesity and insulin resistance [20]. In addition, *CCDC80* had been shown to be secreted by adipocytes and plays dual roles in adipogenesis via the down-regulation of Wnt/β-catenin signaling as well as the induction of *C/EBPα* and *PPARγ* [21]. Our results combined with these previous findings indicate *CCDC80* may promote IMF deposition and may be a key functional gene related to fat regulation. However, the molecular mechanism by which it regulates IMF traits in beef needs to be studied further.

Three clusters of genes positively correlated with IMF deposition were found in our study: *HOXA*, *HOXC*, and *HOXD*. Precise *HOX* gene expression has been shown to be crucial for embryonic patterning [22]. The HOX gene cluster is a highly conserved superfamily of regulatory genes that exists widely in higher eukaryotes and plays a pivotal role in the regulation of cell proliferation, differentiation, migration, and apoptosis [23]. *HOXA1*, *HOXA4*, and *HOXC4* were found in human adipose tissue directly, implicating them in the differentiation of WAT and BAT [24]. A QTL study has identified *HOXA9* [25], and its expression was lower in Holstein–Friesian (IMF, 0.81%) and Hereford (IMF, 1.1%) than in Limousin breed (IMF, 0.53%) [26]. The differences in *HOXA9* expression could be due to differences in muscle type [27]. Compared to the high-IMF (S) and low-IMF (Y, L, and W) groups in the current work, *HOXA9* gene expression was downregulated in this research, with log2FC values greater than eight (Tables S4–6), indicating that the effect of *HOXA9* depends on the muscle fiber composition of muscle location. The accumulation of adipose tissue in differentiated adipocytes is associated with the expression of *HOX* genes [28]. It is likely that the *HOX* gene cluster is involved in physiologic processes in intramuscular adipocytes.

The types and contents of metabolites are closely linked to animal phenotypes. In this study, we identified four key upregulated metabolites in the high IMF group: EA, carcinine, thiostatin and glutaminylglutamic acid. EA (C22:1, n-9) is an unbranched fatty acid and has been shown to cause lipidosis of the myocardium as well as cardiac steatosis in animal feeding experiments [29]. *PPARδ* and its ligand EA had beneficial effects in the treatment of neuroectodermal tumors and Parkinson's disease and EA has potential anti-cancer and neuroprotective effects [30]. *ABCD2*-knockout mice fed a high-EA diet exhibit a rapid expansion of adipose tissue, referred to as adipocyte hypertrophy [31]. In contrast, EA was found to regulate the differentiation of mesenchymal stem cells into osteoblasts/adipocytes by the inhibition of *PPARγ* transcriptional activity [32]. These studies suggested that EA has a dual regulatory role in fat deposition in animals. Carcinine plays a vital role in animal vision as well as in histaminergic neurons in the brain [33]; however, little is known about its effect on meat quality relative to the effects of thiostatin. The addition of dietary glutamine and glutamic acid to piglet rations can accelerate carbon turnover in piglets after weaning [34]. In geese, C16:0, C16:1, and C18:1n9c were positively correlated with intramuscular fat and alanine, and the metabolism of aspartic acid, glutamic acid, d-glutamine, and d-glutamic acid were the major metabolic pathways associated with the flavor of Shaziling pork [35]. The biosynthesis of endogenous fatty acids begins with the synthesis of saturated fatty acids from C2 to C16 mediated by *FASN* [36]. *SCD*, the key rate limiting enzyme in unsaturated fatty acid synthesis, then catalyzes the production of monounsaturated fatty acids from saturated fatty acids, primarily palmitoyl-CoA at 16 carbons and stearoyl-CoA at 18 carbons, resulting in palmitoleoyl-CoA and oleoyl-CoA, respectively [37]. The monounsaturated products of *SCD* are key precursors of triglycerides, and *SCD* is pivotal in fatty acid metabolism [38]. *SCD1*-catalyzed oleic acid, a ligand for *PPAR*γ, has been shown to increase bovine triglyceride levels [39]. The *SCD1* expression level is positively correlated with the marbling score [40]. *SCD5* mutations lead to the excessive deposition of visceral fat, and Wnt, PPAR, C/EBP, and fat synthesis signaling pathways are all affected in zebrafish [41]. HACD is a key catalytic enzyme for the elongation of long-chain fatty acids (LCFAs). The deletion of *HACD2* caused a significant decrease in the synthesis of LCFA above C18 in mouse embryos [42]. In addition, *HACD2* is a candidate gene for the deposition of subcutaneous fat in beef cattle [43]. Studies have shown that *ACOX3* is involved in IMF regulation in broilers [44] and can oxidize straight chain fatty acids in bovine [45]. However, its specific regulatory mechanism is unclear. Based on this previous research and the results of this study, we hypothesize that EA is the key metabolite affecting the IMF content. With respect to its synthesis, carbohydrates are converted to acetyl-CoA by glycolysis and then undergo a series of iterative reactions and elongation by *FASN*. Saturated fatty acid C16 is then catalyzed by SCD1/SCD5 to an unsaturated fatty acid and expanded to C22 (EA) by *HACD2*. EA can be used as a ligand of *PPAR*, and P*PARα*, *PPARβ/δ*, and *PPARγ* are stimulated and act as transcription factors to regulate downstream target genes (such as *FABP4*), accelerate lipid droplet accumulation, promote adipogenesis, and ultimately increase the IMF deposition and improve beef quality. Lipid droplets can also be decomposed by *HSL* into LCFA and VLCFA, and VLCFAs are oxidized to FA by *ACOX3* and then contribute to fatty acid metabolism (Fig. 6G). Our next step is to explore the mechanism by which EA as well as *ACOX3*, *HACD2,* and *SCD5* contribute to the synthesis of triglycerides at the cellular level.

In addition, DEGs and DEMs were enriched in three major KEGG pathways, namely purine metabolism, pyrimidine metabolism, and glycine, serine and threonine metabolism. Some differential metabolites identified in this study were altered in irradiated goat meat, with roles in phenylalanine, tyrosine, and tryptophan biosynthesis and purine metabolism [46]. A lower purine content in meat was significantly associated with a higher abundance of intramuscular fat and marbling [47]. Purine metabolism and the glycine, serine, and threonine pathway were enriched for various differential metabolites in postmortem metabolite analyses of atypical and typical dark, firm, and dry beef [48]. Levels of amino acids promoting sweetness and umami were higher in high IMF beef [49].

## Conclusions

This study revealed a large number of DEGs and DEMs by transcriptome and metabolome analyses of IMF. Of note, IMF is relatively rich in the muscles of Qinchuan cattle bulls and differs substantially with respect to muscle locations as follows: high rib (15.86%), ribeye (14%), striploin (10.44%), and tenderloin (8.67%). We identified *CCDC80* as a candidate gene in the regulation of IMF deposition in beef cattle. In addition, EA, carcinine, thiostatin, and glutaminylglutamic acid were identified as the major metabolites in Qinchuan beef cattle with high IMF levels. IMF deposition could be regulated by the metabolic pathway of unsaturated fatty acids involving the metabolite EA and the genes *ACOX3*, *HACD2,* and *SCD5*. It is possible that the *HOX* gene cluster regulates IMF deposition. In addition, DEGs and DEMs were enriched in three main KEGG pathways, namely purine metabolism, pyrimidine metabolism, and the metabolism of glycine, serine, and threonine. Overall, these findings provide detailed information on the biological mechanism underlying IMF accumulation and support the selection and breeding of Qinchuan cattle.

## Materials and methods

### Animals and sample collection

All procedures were conducted in accordance with the Chinese laws on animal experimentation, approved by the Northwest A&F University’s Experimental Animal Management Committee (EAMC) (protocol number: NWAFUCAST2018-167), and conducted under the authority of the Project License. Four Qinchuan bulls were chosen from the same breeding farm. All cattle were healthy and disease-free and were maintained under the same standard management conditions with free access to feed and water and culled at 24 months of age (associated with the best meat quality for slaughtering). Bulls were fasted for 24 h with free access to water before slaughter and were electrically stunned with a stunner for 5 s, bled, peeled, eviscerated, and split down the midline by a commercial plant (Dingle Yihe Meat Processing Co., Ltd., Xianyang, Shaanxi, China). A set of four muscles (tenderloin, striploin, high rib, and ribeye) were taken immediately after slaughter from an individual animal to determine the IMF content. All other samples (16 samples) were immediately frozen and stored at − 80 °C until the extraction of RNA and metabolites.

### Determination of the IMF content

IMF contents were determined based on the Soxhlet extraction method in China GB/5009.6–2016 “National Food Safety Standard-Determination of Fat in Food”. The specific process was completed by Norminkoda Biotechnology Co., Ltd. (Wuhan, China).

### RNA extraction, sequencing, and transcriptome data analysis

Total RNA extraction, detection of RNA integrity, library construction, and RNA-seq were carried out by Biomarker Technologies Co., Ltd. (Beijing, China). Specifically, the RNA of the muscle samples was extracted, and after the purity, concentration, and integrity of the total RNA were qualified, the library was constructed. After the library passed the quality thresholds, the Illumina NovaSeq6000 sequencing platform was used for paired-end sequencing. The RNA-seq analysis was performed using BMKCloud (www.biocloud.net). Stringent quality control was applied to the data. The clean reads were aligned to the Bos_taurus.ARS_UCD1.2 cattle reference genome (https://bovinegenome.elsiklab.missouri.edu/downloads/ARS-UCD1.2) using the HISAT2 software package (http://www.ccb.jhu.edu/software/hisat2). FPKM [50] (Fragments Per Kilobase of transcript per Million fragments mapped) was used to quantify the expression or transcript level of the gene. Gene expression was analyzed using the DEseq2 package [51]. We defined Fold Change ≥ 2 and FDR < 0.05 as thresholds to obtain differentially expressed genes (DEGs). A Gene Ontology (GO) enrichment analysis and Kyoto Encyclopedia of Genes and Genomes (KEGG) [52] pathway enrichment analysis of DEGs were carried out using the R packages clusterProfiler and topGO.

### LC–MS/MS analysis

The main steps in metabolite extraction include adding an appropriate volume of extraction solution and magnetic beads for grinding, ultrasonic treatment, standing, and centrifugation, collecting the supernatant for vacuum drying, and adding an appropriate amount of extraction solution for reconstitution and testing. The detection platform was the Waters Acquity I-Class PLUS ultra high performance liquid chromatographer in series with the Waters Xevo G2-XS QTOF high resolution mass spectrometer. For peak extraction and alignment, the original data collected by MassLynx V4.2 was processed using the Progenesis QI software package and identified based on the Progenesis QI online METLIN database, a public database, and the self-built library, and theoretical fragment identification was performed at the same time. The precursor ion had a mass deviation of 100 ppm, and the fragment ion mass deviation was less than 50 ppm. Following the qualitative and quantitative metabolite analyses, data quality assessment, annotation, differential expression analysis, and functional enrichment analyses were carried out. Furthermore, extensive data exploration and analysis were performed using the BMKCloud cloud platform (www.biocloud.net). Lastly, metabolites with fold change ≥ 1, Variable Importance in Projection (VIP) ≥ 1, and *P* < 0.05 were chosen as DEMs.

### Statistical analysis

IMF contents are expressed as means ± standard deviations (SD). Means were evaluated by an analysis of variance (ANOVA) with Tukey's tests for multiple comparisons at a significance level of *P* < 0.05 using the GraphPad Prism 9.3 (GraphPad Software Inc., San Diego, CA, USA). PCA and correlation analyses were conducted in R (version 4.0).

## Supplementary Information

**Additional file 1.**

**Additional file 2.**

## Data Availability

The RNA-Seq raw data have been deposited in the Genome Sequence Archive of the National Genomics Data Center, China National Center for Bioinformation/Beijing Institute of Genomics, Chinese Academy of Sciences, under accession number CRA010101 (https://ngdc.cncb.ac.cn/gsa/s/5Dy6cnVO).

## Abbreviations

IMF: Intramuscular fat
EA: Erucic acid
DEG: Differentially expressed gene
DEM: Differentially expressed metabolite
GO: Gene Ontology
KEGG: Kyoto Encyclopedia of Genes and Genomes
VIP: Variable importance in projection
ANOVA: Analysis of variance
PCA: Principal components analysis
WGCNA: Weighted correlation network analysis
ME: Metabolite module
GE: Gene module
