# Supplementary material for: Integrated multi-omics analysis reveals variation in intramuscular fat among muscle locations of Qinchuan cattle
Source: BMC Genomics. 2023 Jul 1;24:367. doi: 10.1186/s12864-023-09452-9 (PMC10314489; doi:10.1186/s12864-023-09452-9)
Supplement: Supplementary file 1 — Additional file 1. [file 12864_2023_9452_MOESM1_ESM.docx]

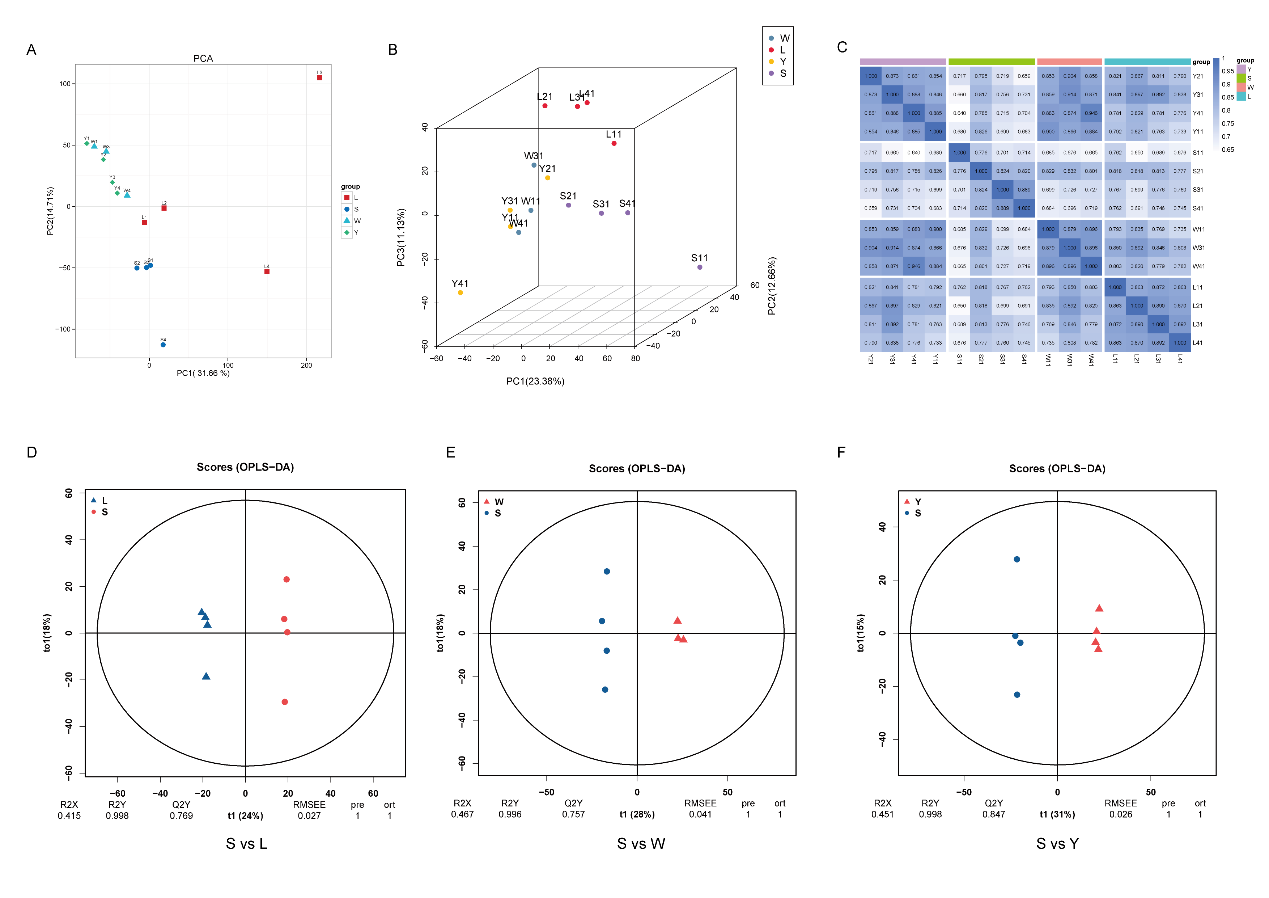
Fig. S1. (A) Principal component analysis (PCA) of the transcriptome of samples. (B) PCA of the metabolome of samples. (C) Correlation analysis between sample metabolomes. Orthogonal projections to latent structures- discriminant analysis (OPLS-DA) between samples: S vs L(D), S vs W groups (E) and S vs Y groups (F). The x-axis represents the difference between groups, and the y-axis represents the difference within groups.


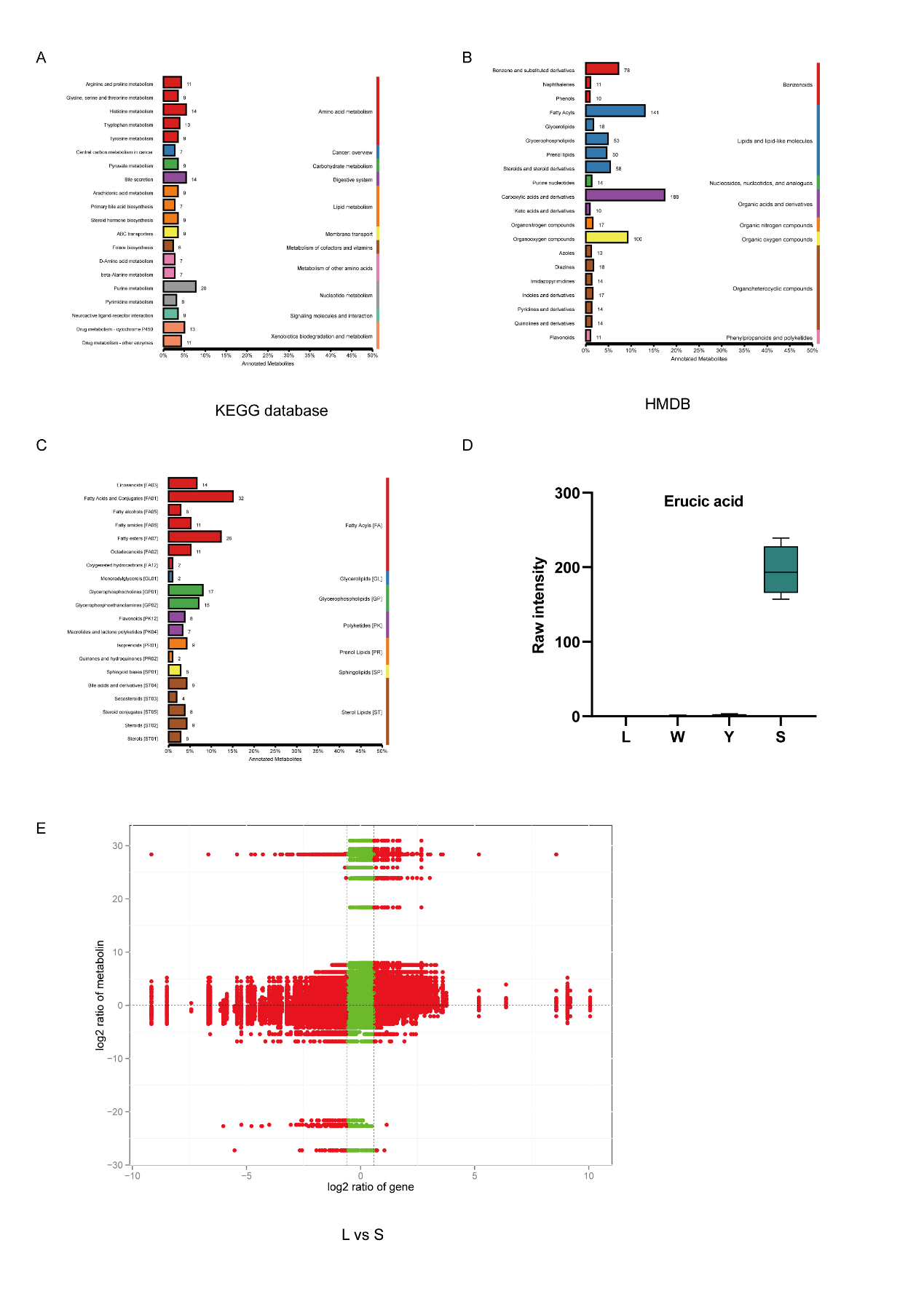


Fig. S2. Metabolites were annotated in KEGG database (A), HMDB (B) and LIPID MAPS (C).

(D) The raw intensity of erucic acid in the four groups. (E) Nine-quadrant diagram of DEGs and DEMs of L vs S. The correlation between all genes and metabolites was calculated based on the Pearson correlation method, and screening was performed according to the correlation coefficient (CC) and correlation coefficient pvalue (CCP). The screening threshold: |CC|>0.80 and CCP<0.05.
